# microRNA-200a silencing protects neural stem cells against cerebral ischemia/reperfusion injury

**DOI:** 10.1371/journal.pone.0172178

**Published:** 2017-02-21

**Authors:** Ji Ma, Shaofeng Shui, Xinwei Han, Dong Guo, Tengfei Li, Lei Yan

**Affiliations:** Department of Interventional Radiology, The First Affiliated Hospital of Zhengzhou University, Zhengzhou, China; Swedish Neuroscience Institute, UNITED STATES

## Abstract

Neural stem cells (NSCs) play major roles in neurological recovery after cerebral infarction (CI). This study was trying to investigate whether miR-200a, a vital regulator in cell proliferation, migration and apoptosis, also has a role in oxygen-glucose deprivation/reperfusion (OGD/R) injured NSCs. In this study, primary NSCs were subjected to OGD/R conditions to mimic an *in vitro* CI model. Before OGD/R induction, NSCs were transfected with vector or shRNA against miR-200a to overexpress or suppress miR-200a expression. The changes in cell viability, apoptosis, migration, the expression of c-Myc, and the phosphorylation of STAT1, STAT3 and MAPK were respectively assessed. Inhibitors of STAT1/3 and MAPK, *i*.*e*., Nifuroxazide and BIRB 796, were used to administrate miR-200a-silenced NSCs, and the expressions of above mentioned proteins were detected. After OGD/R exposure, miR-200a was up-regulated in NSCs (*P* < 0.001). miR-200a silencing alleviated OGD/R-induced the decrease of cell viability and migration (*P* < 0.01); meanwhile, alleviated OGD/R-induced apoptosis via reducing Bax/Bcl-2 ratio and down-regulating p53 and cytochrome c (*P* < 0.01 or *P* < 0.001). c-Myc, p-STAT1, p-STAT3, p-MAPK were all negatively regulated by miR-200a (*P* < 0.01 or *P* < 0.001); more important, the increase of c-Myc induced by miR-200a silencing was abolished by Nifuroxazide or BIRB 796 (*P* < 0.01 or *P* < 0.001). These data indicate miR-200a silencing protects NSCs from OGD/R-induced injury, possibly via regulating the STATs/c-Myc and MAPK/c-Myc signalings.

## Introduction

Cerebral infarction (CI) is one type of ischemic stroke and is the most commonly reported cerebral vascular diseases, accounting for about 70% of strokes [1]. CI can induce several debilitating sequelaes such as hemiplegia, aphasia and dementia, which lead to a serious threat to human health [2]. Thrombolysis or clot-dissolving is the most effective treatment thus far and significantly reduces the risk of long-term dependency on others for daily activities in spite of an increased risk of bleeding in the brain [3]. However, not all patients with a CI are candidates for thrombolysis, since the risk of hemorrhage [2]. Thus, many patients must unfortunately live with long-term neurologic deficits which lead to irreversible brain damage [4]. It will be beneficial to attenuate the extent of CI and cerebral ischemia/reperfusion injury to increase the windows of opportunity for therapeutic interventions [5].

Neural stem cells (NSCs) are cells of the central nervous system that can self-renew and generate the three major neural lineages, *i*.*e*., astrocytes, neurons and oligodendrocytes [6]. After ischemic stroke, adult endogenous NSCs proliferate, differentiate, and migrate from the subventricular zone of the brain to play major roles in neurological recovery [7]. Therefore, a number of NSCs lines are being used in clinical trials for treatment of neurological diseases and strokes, including CI [2, 8].

microRNAs (miRNAs), a family of short noncoding RNA molecules of 20–25 nucleotides, have emerged as critical regulators of cell fate in NSCs during brain development [9]. The miR-200 family comprises five members organized as two clusters, *i*.*e*., miR-200b/200a/429 and miR-200c/141 [10]. It has previously been shown that miR-200 family members play vital roles in the regulation of cell proliferation, cell-cycle exit, differentiation and migration of pluripotent/multipotent stem cells, such as NSCs [11, 12]. Despite the potential importance of miR-200 in understanding NSCs development, the critical link between the miR-200a function and cerebral ischemia/reperfusion injured NSCs is poorly understood. In this paper, we established oxygen-glucose deprivation/reperfusion (OGD/R) model in primary NSCs to mimic an *in vitro* CI milieu. The expression of miR-200a was overexpressed or silenced by transfection with its vector or shRNA. The effects of miR-200a on OGD/R-injured NSCs were assessed by detection the changes in cell viability, apoptosis, migration and c-Myc expression level. Further, to reveal the biological effects of miR-200a, the key factors in STAT and MAPK signaling pathways were also assessed.

## Materials and methods

### NSCs culture

NSCs were isolated from specific pathogen-free grade of C57BL/6 mice (10 individuals, six weeks old, Vital River Laboratories, Beijing, China) as described previously [13]. In brief, mice were killed by cervical dislocation, their cerebral cortex was dissected from the forebrains, after which its cells were digested in trypsin/EDTA solution (0.25% w/v trypsin, 0.02% w/v EDTA, Sigma-Aldrich, St Louis, MO) at room temperature for 15 min. After filtration through a 200-mesh filter and centrifugation at 300 × *g* for 5 min, the cells were cultured in Dulbecco’s modified Eagle’s medium (DMEM)/F12 (Sigma-Aldrich) medium containing 20 ng/mL B27, 20 ng/mL epidermal growth factor (EGF) and 20 ng/mL basic fibroblast growth factor (bFGF) (all from Gibco, Gaithersburg, MD). Cells were maintained at 37°C in a humidified atmosphere under 5% CO_2_. The culture medium was changed every 3 days and the cells were passaged every 7 days. This study was approved by the Animal Ethics Committee of our local hospital and was conducted in accordance with the instructions of our institute.

### OGD/R induction and cell treatment

The OGD/R model was established by exposure of cells cultured in glucose-free medium and were then placed in a modular chamber (MC-101 model, Billups-Rothenberg, Del Mar, CA) filled with gas mixture (1% O_2_, 5% CO_2_, and 94% N_2_) at 37°C. After 2 h, culture medium was changed by the normal medium and cells were cultured under normoxia. Cells cultured under normal conditions were used as a control group.

For suppressing the activation of STAT1/3 and p38 MAPK, 20 μM Nifuroxazide (S4182) and 30 μM BIRB 796 (S1574) (both from Selleckchem, Houston, TX) were respectively added to cells for 24 h before OGD/R induction.

### Cell transfection

Lentivirus expressing shRNAs against either miR-200a (sh-miR-200a group) or scrambled hairpin sequence (sh-scramble group) (GeneChem, Shanghai, China) was used to infect cells with the addition of 8 μg/mL polybrene (Invitrogen, Carlsbad, CA). miR-200a expression plasmid (miR-200a group) or its scrambled control (scramble group) (GeneChem) was transfected to cells by using Lipofectamine 2000 (Invitrogen) following the manufacturer’s protocol. After 48 h, cells were collected for the use of the subsequent experiments.

### qRT-PCR

The miRNAs in cells were extracted by miRNeasy Mini Kit (Qiagen, Shenzhen, China). Reverse transcription was performed using 1 μg of total miRNAs and the primers specific to miR-200a (5’-GTC GTA TCC AGT GCA GGG TCC GAG GTA TTC GCA CTG GAT ACG ACA CAT CGT-3’) or the U6 RNA internal control (Fw: 5’-CTC GCT TCG GCA GCA CA-3’ and Rv: 5’-AAC GCT TCA CGA ATT TGC GT-3’) by PrimeScript Reverse Transcriptase (Takara, Dalian, China) [14]. qRT-PCR was conducted on a QuantStudio 6 Flex Realtime PCR system (Applied Biosystems, Carlsbad, CA). Data were analyzed by using the classic 2^−ΔΔCT^ methods.

### Cell viability

Transfected cells were seeded in 96-well plates with a density of 5 × 10^3^ cells/well for adherence, and then were exposed to OGD/R conditions. After 24 h, 20 μL CCK-8 reagent (Dojindo Molecular Technologies, Dojindo, Japan) was added, and the plates were incubated at 37°C for 4 h. After shaking gently for 10 min, the absorbance was detected at 450 nm using a Microplate Reader (Bio-Rad, Hercules, CA, USA).

### Apoptosis assay

Quantification of apoptotic cells were performed by Annexin V-FITC/PI apoptosis detection kit (4 A Biotech, Beijing, China) according to the manufacturer’s instructions. Briefly, after OGD/R exposure, the transfected cells were re-suspended in 200 μL binding buffer containing 10 μL Annexin V-FITC and 5 μL PI. After incubation at room temperature for 30 min in the dark, FITC-positive and PI-negative cells were immediately distinguished using flow cytometry (Becton Dickinson, Mountainview, CA).

### Cell migration assay

After the transfected cells exposed to OGD/R conditions, cells migration was determined using Transwell migration chambers (8.0-μm pore, Costar-Corning, NY). The upper chamber was filled with cells which were suspended in serum-free medium, and the lower chamber was filled with complete medium as a chemoattractant. After incubation at 37°C for 24 h, the non-migrated cells on the upper compartment were wiped off by cotton swabs. The migrated cells were stained with crystal violet (Beyotime, Nantong, China) and counted with a light microscopy.

### Western blot

The total protein of cells was extracted using RIPA lysis buffer (Beyotime) and the protein concentration was quantified by BCA^™^ Protein Assay Kit (Pierce, Appleton, WI) according to the manufacturers’ instructions. The proteins were resolved over 10–12% sodium dodecyl sulfate-polyacrylamide gel electrophoresis (SDS-PAGE) and transferred to a polyvinylidene fluoride (PVDF) membrane. After blocking in 5% non-fat dry milk for 1 h at room temperature, the membranes were incubated at 4°C overnight with antibodies against Bcl-2, Bax, p53, cytochrome c, c-Myc, p-STAT1, STAT1, p-STAT3, STAT3, p-MAPK, MAPK and GAPDH (dilution 1:1000, Santa Cruz Biotechnology, Santa Cruz, CA). The membranes were then incubated with the HRP-conjugated second antibodies for 1 h at room temperature. Blots were visualized by EasyBlot ECL Kit (Sangon Biotech, Shanghai, China) and the intensity of the blots was quantified by ImageJ 1.49 (National Institutes of Health, Bethesda, MD).

### Statistical analysis

All data were from three independent experiments and represented mean ± standard derivations (SD). Statistical significance between different groups was analyzed by one-way analysis of variance (ANOVA) with Duncan’s multiple range tests in SPSS 20 (IBM, NY). *P* < 0.05 was considered to indicate statistical significance.

## Results

### NSCs injury after OGD/R is accompanied with miR-200a up-regulation

**Fig 1:**
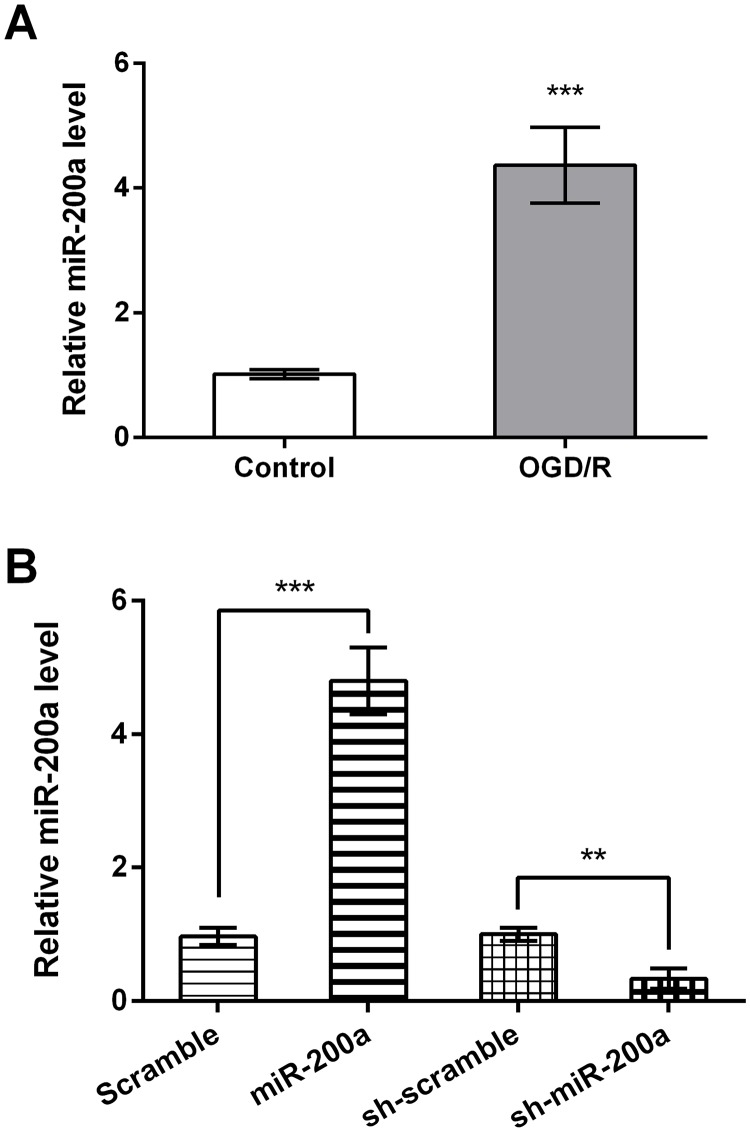
NSCs injury after OGD/R is accompanied with miR-200a up-regulation. (A) Primary NSCs were exposed to OGD/R conditions, and the expression level of miR-200a was then measured by qRT-PCR analysis. (B) NSCs were transfected with vector or shRNA against miR-200a, and the transfection efficiency was verified by detection of miR-200a expression also using qRT-PCR. ** *P* < 0.01. *** *P* < 0.001.

Firstly, the expression level of miR-200a in NSCs was measured by qRT-PCR after OGD/R exposure. As results showed in Fig 1A, miR-200a was notably up-regulated in NSCs after OGD/R induction (*P* < 0.001). This data indicated miR-200a might be implicated in the OGD/R-induced injury in NSCs. Then, miR-200a expression in cells were increased or suppressed by transfected with vector or shRNA against miR-200a, and the transfection efficiency was verified *in vitro*. As data given in Fig 1B, miR-200a-overexpressed and -silenced cells were successfully generated (*P* < 0.01 or *P* < 0.001).

### miR-200a silencing protects NSCs against OGD/R-induced injury

**Fig 2:**
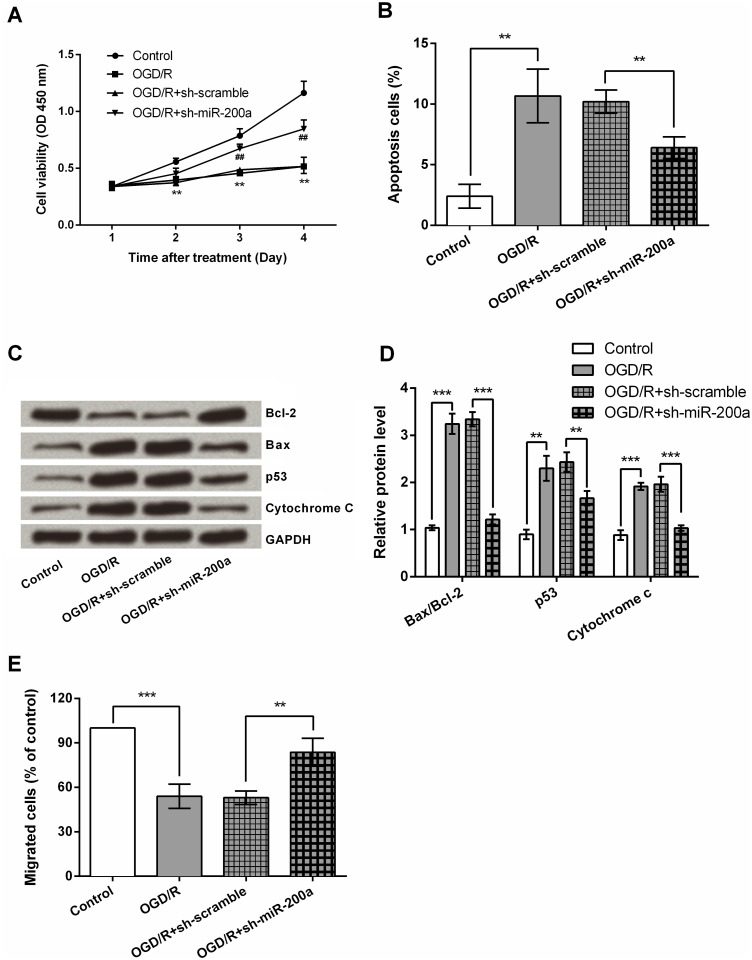
miR-200a silencing protects NSCs against OGD/R-induced injury. NSCs were transfected with shRNA against miR-200a or its negative control (scrambled), and then were exposed to OGD/R conditions. (A) Cell viability, (B) apoptotic cells population, (C) and (D) the expression of apoptosis-related factors, and (E) cell migration were respectively assessed by MTT, flow cytometry, Western blotting and Transwell assay. ** *P* < 0.01. *** *P* < 0.001. ^##^ *P* < 0.01 versus OGD/R + sh-scramble group.

To determine the role of miR-200a in NSCs, miR-200a-silenced cells were exposed to OGD/R conditions. As shown in Fig 2A, OGD/R significantly reduced cellular viability (*P* < 0.01), while this effects was partly abolished by miR-200a silencing (*P* < 0.01). OGD/R-injured NSCs exhibited notable increases in apoptotic cells population, Bax/Bcl-2 ratio, as well as p53 and cytochrome c expression levels (*P* < 0.01 or *P* < 0.001), and these increases were also abolished by miR-200a silencing (*P* < 0.01 or *P* < 0.001, Fig 2B–2D). Moreover, the decrease of migrated cells number caused by OGD/R was recovered by miR-200a silencing (*P* < 0.01, Fig 2E). Altogether, these data indicated a protective role of miR-200a in OGD/R-induced injury in NSCs.

### c-Myc is negatively regulated by miR-200a

**Fig 3:**
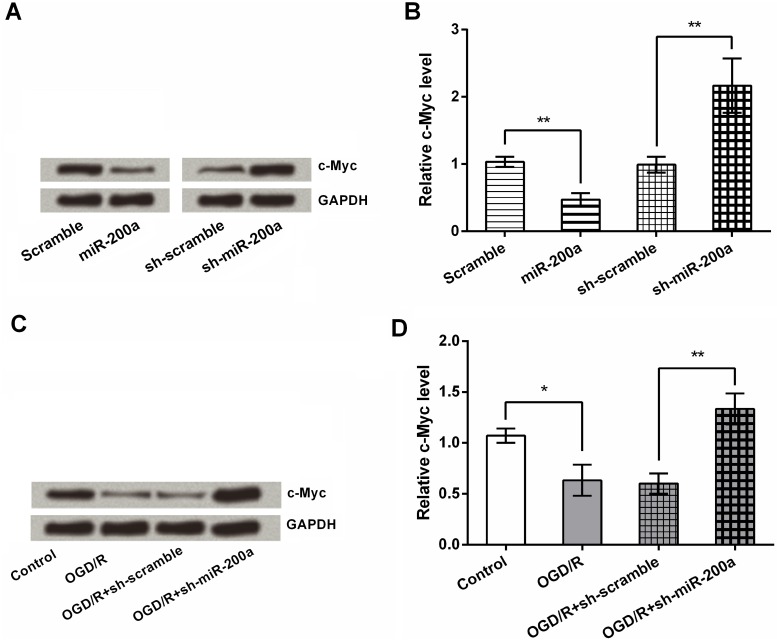
c-Myc is negatively regulated by miR-200a. (A) NSCs were transfected with shRNA against miR-200a or its negative control (scrambled), and the expression changes in c-Myc were determined by Western blotting. (B) After transfection, the modified NSCs by miR-200a were exposed to OGD/R conditions, and then the expression level of c-Myc was assessed again. * *P* < 0.05. ** *P* < 0.01.

c-Myc is a pivotal regulator in cell proliferation, survival, genetic instability, apoptosis and migration [15, 16]. We therefore investigated if the functional effects of miR-200a silencing on NSCs were correlated with c-Myc expression. c-Myc down-regulation was observed in miR-200a-overexpressed cells, while up-regulation in miR-200a-silenced cells (*P* < 0.01, Fig 3A and 3B), indicating c-Myc was negatively regulated by miR-200a. Furthermore, the protein expression of c-Myc was down-regulated after OGD/R (*P* < 0.05) and this down-regulation was abolished by miR-200a silencing (*P* < 0.01, Fig 3C and 3D). These preliminary data indicated that c-Myc might be implicated in miR-200a-mediated OGD/R injury in NSCs.

### miR-200a inhibits c-Myc involved in STAT and MAPK signaling pathways

**Fig 4:**
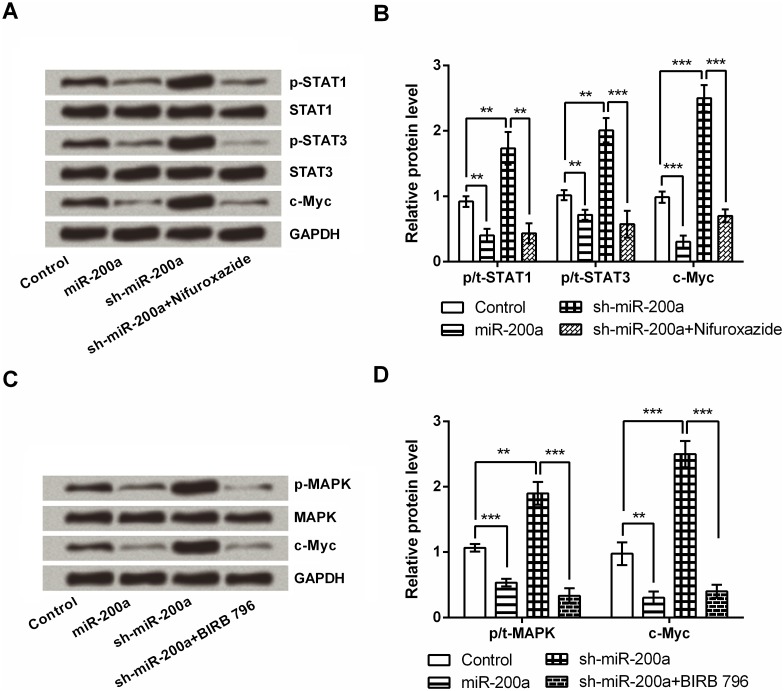
miR-200a inhibits c-Myc involved in STAT and MAPK signaling pathways. (A) and (B) NSCs were transfected with vector or shRNA against miR-200a, and then the miR-200a-silenced cells were treated with or without Nifuroxazide. p-STAT1, STAT1, p-STAT3, STAT3 and c-Myc expression levels were determined by Western blotting. (C) and (D) NSCs were transfected with vector or shRNA against miR-200a, and then the miR-200a-silenced cells were treated with or without BIRB 796. p-MAPK, MAPK and c-Myc expression levels were also detected by Western blotting. ** *P* < 0.01. *** *P* < 0.001.

c-Myc has been reported as a downstream gene of STAT and MAPK signaling [17]; therefore Nifuroxazide (STAT1/3 inhibitor) and BIRB 796 (p38 MAPK inhibitor) were used in this study to detect the possible mechanisms of c-Myc-dependent miR-200a regulation. As shown in Fig 4A–4D, miR-200a significantly inactivated STAT1, STAT3, MAPK and down-regulated c-Myc (*P* < 0.01 or *P* < 0.001); conversely, miR-200a silencing affected these protein expressions resulted in completely opposite impacts (*P* < 0.01 or *P* < 0.001). More importantly, the regulatory effects of miR-200a silencing on these proteins were respectively abolished by the presence of Nifuroxazide or BIRB 796 (*P* < 0.01 or *P* < 0.001).

## Discussion

As various classes of miRNAs are emerging as the master controllers of transcription and translation, their functional changes may be factors in deciding the pathological outcome after stroke [18]. Investigating the mechanism of action of miRNAs has become a hot topic which will open new avenues for therapeutically targeting CI [19]. In this study, we have demonstrated that miR-200a was up-regulated in NSCs after OGD/R exposure. miR-200a silencing significantly alleviated OGD/R-induced injury via improving cell viability and migration, and suppressing apoptosis. c-Myc was negatively regulated by miR-200a with or without exposure to OGD/R conditions. Furthermore, STAT and MAPK signaling pathways were both activated by miR-200a silencing, and blocking these two signaling by using their corresponding inhibitors abolished the regulatory effects of miR-200a silencing on c-Myc expression.

Recent studies showed that CI alters miRNA expression profiles in rodents as well as in humans [19]. For instance, miR-200a was up-regulated early after ischemic preconditioning [20], this was also confirmed in our study. Of note, the miRNAs responds rapidly and many of the changes sustain through the progression of the brain damage following IC [19, 21]. *In vitro* study has revealed the neuroprotective effects of miR-200a by increasing cell survival [20]. In line with this previous study, we demonstrated that knocking down of miR-200a remarkably attenuated OGD/R-induced injury by modulation cell viability, apoptosis and migration, indicating a protective role of miR-200a in the postischemic NSCs.

c-Myc is a multifunctional protein that regulates a wide variety of cellular processes, such as cell growth, cell-cycle progression and metabolism, differentiation, apoptosis and cell motility [22]. It has been reported that miR-200 family including miR-200a can up-regulated the c-Myc-mediated transcription [23]. Functional studies validated increased c-Myc activity as a potent contributor to the enhanced proliferation and self-renewal of NSCs [24]. In accordance with previous reports, we demonstrated that c-Myc was negatively regulated by miR-200a, which might indicate miR-200a exhibited neuroprotective effects via regulating c-Myc.

Many molecular and cellular signaling factors modulate neuronal homeostasis in the ischemic brain [25]. Among these signaling molecules, MAPKs and STATs are considered to be involved in mitochondria dysfunction, which causes the cellular apoptosis or necrosis [26]. Activation of STAT1 and STAT3 leads to target gene transcription, making these factors essential drivers of cellular proliferation [27]. Sustained activation of MAPK has been shown to be associated with neuronal apoptosis, and selective MAPK inhibitors have been shown to promote survival [28]. In the current study, we demonstrated that STAT1, STAT3 and MAPK were all inactivated by miR-200a; therefore, we inferred miR-200a protected NSCs might be via regulating STAT1/3 and MAPK. More important, the up-regulation of c-Myc induced by miR-200a silencing was abolished by STAT1/3 and MAPK inhibitors. In fact, previous studies have indicated the correlation between STATs, MAPK and c-Myc [29, 30]; however, the hypothesis of miR-200a functions NSCs via regulating STATs/c-Myc and MAPK/c-Myc signaling requires further verification.

To summarize, our data highlight miR-200a silencing protects NSCs from OGD/R-induced injury, possibly via regulating the STATs/c-Myc and MAPK/c-Myc signaling. This study will be helpful for facilitating the development of therapeutic agents for the treatment of CI.
